# Resuscitative endovascular balloon occlusion of the aorta may contribute to improved survival

**DOI:** 10.1186/s13049-020-00757-2

**Published:** 2020-06-30

**Authors:** Makoto Aoki, Toshikazu Abe, Shuichi Hagiwara, Daizoh Saitoh, Kiyohiro Oshima

**Affiliations:** 1 Department of Emergency Medicine, Gunma University Graduate School of Medicine, Maebashi, Japan; 2 Department of General Medicine, Juntendo University, Tokyo, Japan; 3 Health Services Research and Development Center, University of Tsukuba, Tsukuba, Japan; 4 Department of Emergency Medicine, National Hospital Organization Takasaki General Medical Center, Takasaki, Japan; 5 Department of Traumatology and Emergency Medicine, National Defense Medical College, Tokorozawa, Japan

**Keywords:** Resuscitative endovascular balloon occlusion of the aorta (REBOA), Japan, Logistic models, Mortality trend, Trauma

## Abstract

**Background:**

Resuscitative endovascular balloon occlusion of the aorta (REBOA) is an increasingly used trauma resuscitation procedure, however, there are no reports of whether or not the survival of patients treated with REBOA increases over time.

**Methods:**

This retrospective cohort study from a nationwide trauma registry in Japan was conducted between 2004 and 2015. Patients treated with REBOA were divided into three calendar year periods: early-period (2004–2007), mid-period (2008–2011), and late-period (2012–2015). The primary outcome of in-hospital survival was compared between the periods (early-period: reference) using mixed effects logistic regression analysis after adjustment for characteristics, trauma severity, and therapeutic choices.

**Results:**

Of 236,698 trauma patients, 633 patients treated with REBOA were analyzed. Distribution of the patients across periods was as follows: early-period (91), mid-period (276), and late-period (266). In-hospital survival was 39, 49, and 60% in the early-period, mid-period, and late-period, respectively. In regression modeling, the late-period (OR = 2.976, 95% CI = 1.615–5.482) was associated with improved in-hospital survival compared to the early-period, however, the mid-period (OR = 1.614, 95% CI = 0.898–2.904) was not associated with improved survival.

**Conclusions:**

Survival of patients treated with REBOA during the late-period improved compared with survival during the early-period, after adjustment for characteristics, trauma severity, and therapeutic choices. REBOA may be one of the important factors related to progression of modern trauma treatment.

## Background

Resuscitative endovascular balloon occlusion of the aorta (REBOA) is a worldwide topic in modern trauma care. The first use of REBOA was reported more than 50 years ago [1]. The clinical application of REBOA originated from treatments for abdominal aneurysm rupture [2] and obstetric complications [3], and has recently extended into trauma medicine [4, 5]. In Japan, REBOA was approved by the Japanese Ministry of Health in 2000 and initially used as an alternative method to resuscitative open aortic cross-clamping [6] in trauma medicine, after which it became widely recognized.

Nationwide multi-institutional studies reported that REBOA for severe trauma patients was associated with higher mortality [7, 8], ranging from 36 to 76% [7–9]. Through the development of advances in trauma practices, survival of severely injured patients has improved in Japan [10–12], however, there is no evidence that long-term survival of REBOA patients has improved. Therefore, this study aimed to investigate whether survival of patients treated with REBOA improved during a 12–year period.

## Methods

### Study design

This retrospective cohort study was conducted to evaluate whether survival of severe trauma patients treated with REBOA was improved during the time period of 2004 to 2015 or not, using recorded data from the Japan Trauma Data Bank (JTDB). The study was approved by the medical ethics committee of the Gunma University Hospital.

### Data collection

Data were obtained from the JTDB, a nationwide trauma registry established in 2003 by the Japanese Association for the Surgery of Trauma and the Japanese Association for Acute Medicine to improve and ensure the quality of trauma care in Japan. During the study period, a total of 260 hospitals including 95% of tertiary emergency medical centers in Japan participated in the JTDB. The JTDB collects 92 data elements that are related to patient and hospital information such as patient demographics, injury type, transport type, prehospital treatment, prehospital vital signs, vital signs at hospital arrival, trauma scores such as abbreviated injury scale (AIS) score for head, chest, abdomen and pelvis, injury severity score (ISS), revised trauma score (RTS), probability of survival (Ps), in-hospital procedures, blood transfusion quantity within 24 h from arrival at hospital, in-hospital mortality. However, detailed information about the use of REBOA, such as indication, occlusion time, and placement zone are not recorded in the JTDB.

### Patient selection

Patients treated with REBOA were included in the study. Study patients were defined after the following exclusion criteria: dead on arrival, an AIS score = 6 (i.e., unsurvivable injury) for any region, an unclear AIS score for any region, aortic cross clamping, cardiopulmonary resuscitation (CPR), and missing data on patient survival. The existence of aortic cross clamping and CPR were thought to be indicative of resuscitation and strongly related to outcome, therefore, patients with those procedures were excluded. Study patients were analyzed according to three calendar year periods: early-period (2004–2007), mid-period (2008–2011), and late-period (2012–2015).

### Study endpoints

The primary outcome of this study was in-hospital mortality, and the secondary outcome was survival during the first 2 days because REBOA was generally used as a resuscitation procedure in acute trauma phase.

### Statistical analyses

Data collected in this study contained missing values, especially for prehospital vital signs. There is no established statistical method to identify the reasons for missing values, however, the occurrence of missing values in this study was considered to be strongly related to the other observed variables from a clinical perspective, and the reason for missing values was assumed to be random. Therefore, missing data on the collected variables were completed by multivariate imputation by chained equations, and 20 datasets were produced.

After pooling all the imputed datasets into one dataset, continuous variables were expressed as medians (interquartile range [IQR]), and categorical variables were presented as counts and percentages. Comparisons of continuous variables between three calendar year periods were performed using the Kruskal–Wallis test. Categorical variables were expressed as counts and percentages, and comparisons of each categorical variable between periods were performed using the Chi-Square test and/or Fisher’s exact test. Predictive values were integrated across the imputed datasets, based on Rubin’s rule [13].

To assess whether in-hospital survival was improved over time, univariate analyses and mixed-effects logistic regression analyses were performed. Calendar year periods were included as a categorical variable, with the early-period as the reference period. We carefully selected confounders on the assumption that none was directly affected by the calendar year periods. Issues with variable multicollinearity were assessed by variance inflation factor (VIF) analysis with the tolerance value set at < 2. The covariates included age, gender, injury type, transport type, prehospital vital signs (i.e., prehospital systolic blood pressure, prehospital heart rate and prehospital respiratory rate), vital signs at hospital arrival (i.e., systolic blood pressure, heart rate, respiratory rate and Glasgow Coma Scale [GCS] value), AIS scores for head, chest, abdomen, and pelvis, and focused assessment with sonography for trauma (FAST). In addition, mixed-effects logistic regression analysis was performed with adjustment for patient demographics (such as age and gender), trauma severity (such as injury type, prehospital vital signs, vital signs at hospital arrival, and AIS score), therapeutic choices (such as transporter type and FAST), and calendar year on outcome. In the analyses, patient demographics, trauma severity, therapeutic choices, and calendar year were considered fixed effect variables, and the hospital’s unique identifier was considered a random effect variable. This model simultaneously adjusted for both patient level and within-hospital level confounding. Regarding in-hospital survival, we performed sub-group analyses (severe traumatic brain injury (AIS for head≧3), severe abdominal injury (AIS for abdomen≧3) and severe pelvic injury (AIS for pelvis≧3)).

Next, we performed analyses with a model in which calendar year was incorporated as a continuous variable, and a mixed-effects logistic regression model was used for in-hospital survival. Association between calendar year and outcome was plotted by a generalized additive mixed model, which was fit using the residual maximum likelihood method to account for possible nonlinear relationships between calendar year and outcome. The model was also adjusted for patient demographics such as age and gender, trauma severity such as injury type, prehospital vital signs, vital signs at hospital arrival, AIS score, therapeutic choices such as transporter type and FAST, and the hospital’s unique identifier. Calendar year was incorporated into the models as a continuous variable and a smoothing term. We performed sensitivity analysis by univariate linear regression to find trends between in-hospital survival and calendar year. We performed additional analysis to evaluate whether survival of the patients who underwent CPR and treated REBOA were improved.

Second, to assess whether the use of REBOA was associated with improvement of in-hospital survival among severely injured patients, we set matched patients without REBOA by using propensity score matching (PSM). A logistic regression analysis was performed to estimate a PS for the prediction of REBOA+ or REBOA- based on available predictors. Clinically important confounders were included in the calculation of the PS. The variables of PSM were age, sex, calendar year periods, injury type, transport type, prehospital vital signs, vital signs at hospital arrival, AIS code for head, chest, abdomen and pelvis, and abdominal focused assessment sonography for trauma (FAST), initial treatments and blood transfusion quantity within first 24 h. The PSM extracted 1:1 matched pairs of patients using a caliper with 0.2 with REBOA+ or REBOA- based on the averaged PS. The absolute standardized difference of variables for the estimation of PS was used to assess the match balance, whereby an absolute standardized difference > 0.1 represented a meaningful imbalance. In the PS-matched cohorts, univariate analyses were performed to evaluate outcome between REBOA+ and REBOA-.

Statistical significance was defined as a two-sided *p* value < 0.05 or was assessed by a 95% confidence interval (CI) in all statistical analyses. Statistical analyses were performed by the IBM SPSS Statistics version 23.0 (SPSS Inc., Chicago, Illinois, USA) and R software (version 3.5.2; R Foundation for Statistical computing, Vienna, Austria). Multiple imputation was performed with R package “mice”, mixed-effects logistic regression analysis modeling was performed with R package “lme4”, generalized additive mixed modeling was performed with R package “mgcv” and propensity score matching was performed with R package “Matching”.

## Results

**Fig. 1:**
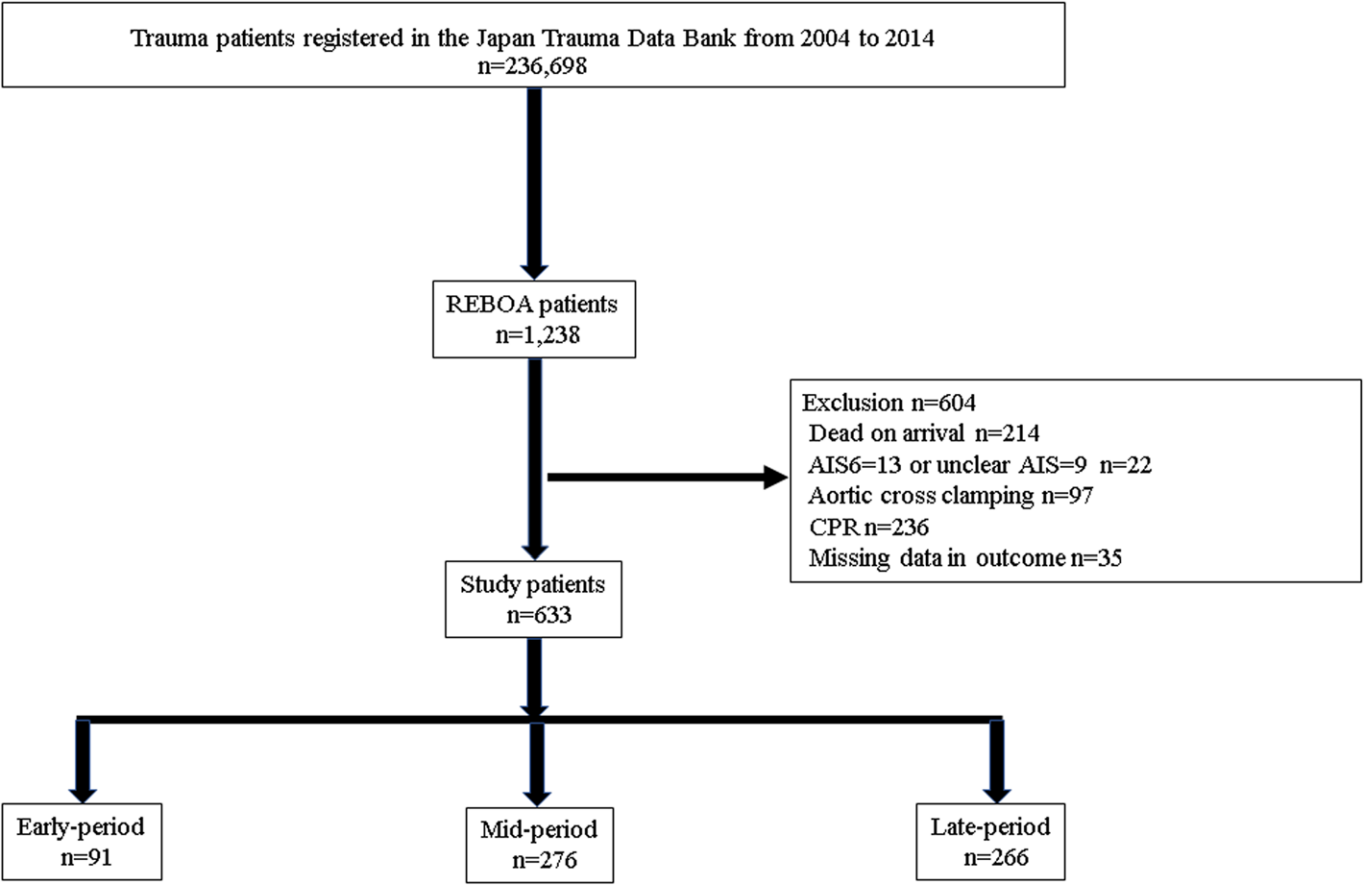
Flow chart of patients included in this study. REBOA, resuscitative endovascular balloon occlusion of the aorta; AIS, abbreviated injury scale; CPR, cardiopulmonary resuscitation

A total of 236,698 patients were registered in the JTDB from 1 January 2004 to 31 December 2015 (Fig. 1), of whom 1238 were treated with REBOA. From these 1238 patients, 604 were excluded for the following reasons: dead on arrival (214), AIS score = 6 (13), unclear AIS score (9), aortic cross clamping (97), CPR (236), and missing data in primary outcome (35). Therefore, a total of 633 patients treated with REBOA were analyzed.

Characteristics of the multiply imputed dataset are shown in Table 1, and the distribution of naïve data and proportions of missing values are shown in Supplemental Table 1. The distribution of patients across the periods was as follows: early-period, *n* = 91; mid-period, *n* = 276; and late-period, *n* = 266. The median age of patients was 54 (34–71) years, and the majority of patients were male (66%). The most common injury type was traffic accident (60%). Regarding transport type, physician treatment tended to be performed in the late-period compared to the early-period (*p* < 0.001). While, there was no difference in prehospital intravenous fluids administration (*p* = 0.137). There was significant difference in prehospital systolic blood pressure (*p* = 0.046), however, there were no significant differences in other prehospital vital signs and vital signs at hospital arrival. There were no significant differences in trauma scores (AIS for head, chest, abdomen and pelvis, ISS, RTS and Ps). The percentages of patients who had a positive FAST assessment were significantly higher in the early-period (*p* = 0.029). Regarding treatment types, the percentage of patients who had a celiotomy was significantly higher in the early-period (*p* = 0.026). There was no significant difference in blood transfusion quantity (*p* = 0.218) across the periods.

**Table 1:** Characteristics of multiply imputed dataset

Subgroups	Early-period (2004–2007) (*n* = 91)	Mid-period (2008–2011) (*n* = 276)	Late-period (2012–2015) (*n* = 266)	*P*-Value
Age	49 (30–68)	54 (32–7)	57 (36–72)	0.136
Sex, Male (%)	64 (70)	178 (65)	177 (67)	0.587
Injury Type				0.987
TA	56 (61)	162 (59)	162 (61)	
Fall	24 (26)	72 (26)	69 (26)	
Other blunt	5 (5.7)	21 (7.6)	16 (6.0)	
Penetrate	6 (6.9)	21 (7.6)	19 (7.2)	
Transport type				0.001
Ambulance	83 (91)	223 (81)	186 (70)	
Dr-car	2 (2.2)	10 (3.6)	26 (9.8)	
Helicopter	6 (6.8)	41 (15)	53 (20)	
Other	0 (0)	2 (0.7)	1 (0.4)	
Prehospital treatment				
Intravenous fluids	5 (5.5)	32 (11.6)	35 (13.2)	0.137
Prehospital vital signs				
sBP	98 (78–118)	94 (78–118)	104 (80–129)	0.046
HR	96 (79–120)	96 (80–120)	100 (82–120)	0.331
RR	24 (18–30)	24 (21–30)	24 (20–30)	0.064
Vital signs at hospital arrival				
sBP, mmHg	80 (40–104)	80 (62–105)	80 (62–111)	0.452
HR	108 (93–126)	105 (85–124)	109 (85–128)	0.602
RR	24 (20–30)	27 (20–30)	25 (20–30)	0.183
GCS	9 (3–14)	11 (6–14)	12 (6–14)	0.617
AIS, n, median (95% CI)				
Head	41, 3 (3–5)	105, 4 (3–5)	100, 4 (3–5)	0.784
Chest	52, 4 (3–4)	162, 4 (3–4)	166, 4 (3–4)	0.281
Abdomen	71, 4 (3–4)	204, 3 (3–4)	175, 4 (3–4)	0.381
Pelvis	51, 3 (2–5)	171, 4 (3–5)	175, 4 (3–5)	0.529
ISS	33 (20–45)	34 (20–45)	34 (22–45)	0.610
RTS	5.4 (3.6–6.9)	5.9 (4.2–7.1)	6.0 (4.4–7.1)	0.125
Ps	55 (13–92)	63 (23–89)	58 (23–89)	0.734
Abdominal FAST				0.029
Positive	59 (65)	171 (62)	132 (50)	
Negative	29 (32)	97 (35)	124 (47)	
Not conducted	3 (3.3)	8 (3.0)	10 (3.8)	
Initial Treatment				
Thoracotomy	3 (3.3)	16 (5.8)	13 (4.9)	0.632
Celiotomy	52 (57)	143 (52)	114 (43)	0.026
TAE	25 (28)	69 (25)	89 (34)	0.090
Blood Transfusion Quantity	24 (16–40)	26 (16–44)	22 (14–38)	0.218

*TA* traffic accident, *sBP* systolic blood pressure, *HR* heart rate, *RR* respiratory rate, *GCS* Glasgow Coma Scale, *AIS* abbreviated injury scale, *ISS* injury severity score, *RTS* revised trauma score, *Ps* provability of survival, *FAST* focused assessment with sonography for trauma, *TAE* transcatheter arterial embolization

**Fig. 2:**
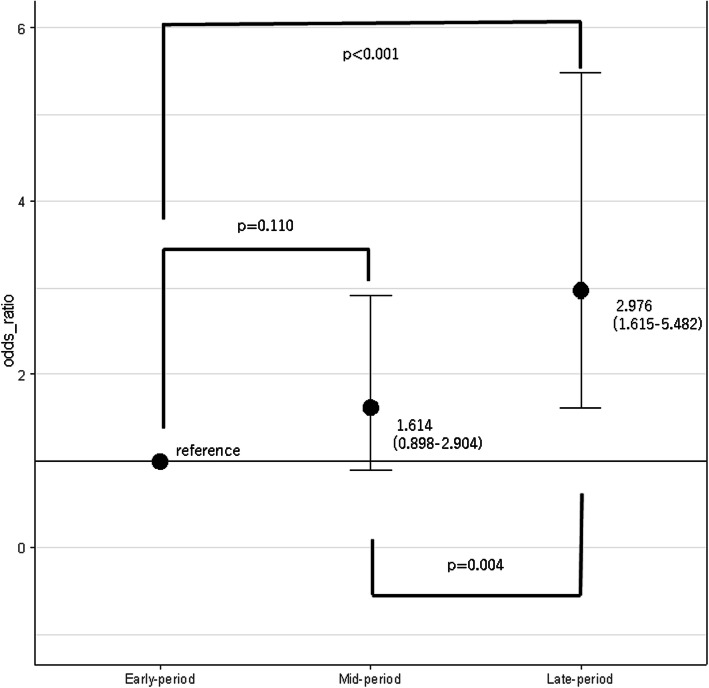
Comparison of in-hospital survival at each calendar year period. *The model was adjusted for age, gender, injury type, transport type, vital signs at hospital arrival (i.e., systolic blood pressure, heart rate, respiratory rate, and Glasgow Coma Scale [GCS] value), AIS score for head, chest, abdomen, and pelvis, FAST, and the hospital’s unique identifier. AIS, abbreviated injury scale; FAST, focused assessment with sonography for trauma

**Fig. 3:**
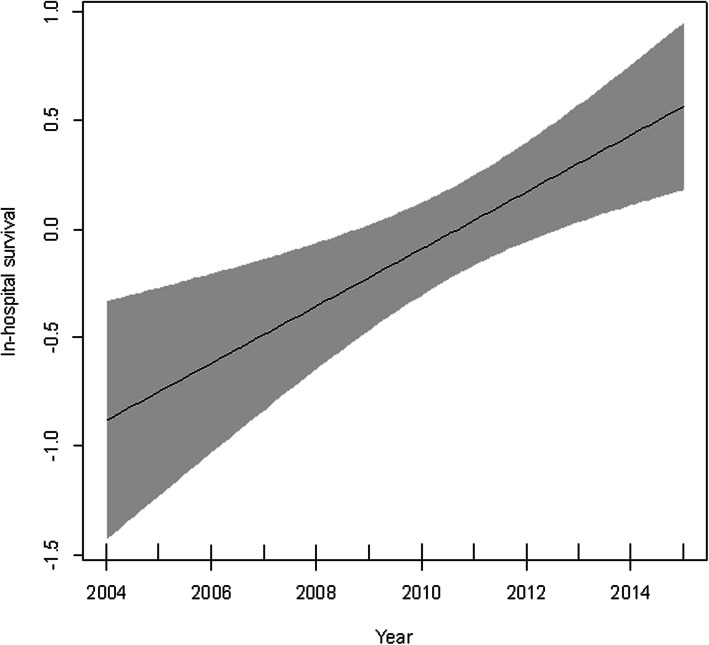
Association between calendar year and in-hospital survival. The shaded region represents the 95% confidence intervals for the estimated points. The model was adjusted for age, gender, injury type, transport type, vital signs at hospital arrival (i.e., systolic blood pressure, heart rate, respiratory rate, and Glasgow Coma Scale [GCS] value), AIS score for head, chest, abdomen, and pelvis, FAST, and the hospital’s unique identifier. AIS, abbreviated injury scale; FAST, focused assessment with sonography for trauma

From comparison of outcomes between the calendar year periods, in-hospital survival was 39, 49, and 60% in the early-period, mid-period, and late-period, respectively (Table 2). Survival during the first 2 days was 43, 57, and 71% in these groups, respectively. Subgroups’ and matched patients’ outcome were shown in Supplemental Table 2. Outcome assessed by mixed effects logistic regression modeling demonstrated that the late-period (Odds ratio (OR) = 2.976, 95% CI = 1.615–5.482) was associated with improved in-hospital survival compared to the early-period, however, the mid-period (OR = 1.614, 95% CI = 0.898–2.904) was not associated with improved survival (Fig. 2). When calendar year was incorporated into a generalized additive mixed model as a continuous variable, it was significantly associated with increased in-hospital survival (*p* < 0.001) (Fig. 3). This result revealed an increasing monotonic association between calendar year and in-hospital survival. Sensitivity analysis demonstrated a monotonic trend between in-hospital survival and calendar-year, and the annual increase in survival percentage was 3.0% (Supplemental Table 3). On the other hands, the survival of the patient who had CPR and were treated by REBOA was not improved (Supplemental Table 4).

**Table 2:** Outcome of REBOA patients according to year groups

Outcome	2004–2007	2008–2011	2012–2015	P-Value
In-hospital survival	35 (39)	134 (49)	160 (60)	0.001
Survival during the first 2 days	39 (43)	156 (57)	190 (71)	< 0.001

Missing numbers, Survival during the first 2 days = 2

*REBOA* resuscitative endovascular balloon occlusion of the aorta

## Discussion

This study from JTDB demonstrated that survival of patients who were treated with REBOA during the late-period (2012–2015) was increased compared with survival during the early-period (2004–2007). While clinical severity and indication for REBOA chronologically changed, the survival of REBOA patients was improved in the late-period (2012–2015) after adjustment for confounders.

### Comparison with previous study

The previous report [10] from JTDB pointed out the problem that mortality of severely injured patients who needed definitive interventions was not decreased until 2011. Subsequently, reports since 2010 have showed that mortality of severely injured patients with Ps < 0.5 or ISS > 16 has decreased [11, 12]. The present study adds information about the progress of trauma care in Japan. For example, the survival of severely injured patients who were treated with REBOA was still not improved into the mid-period (2008–2011 years), however, it was achieved in the late-period (2012–2015 years). To our knowledge, this is the first report that investigated the outcome of patients who were treated with REBOA for a long period of time.

We cannot simply compare the REBOA patients’ survival with other reports. However, even if we considered trauma severity of patients, indication for REBOA use, and reported time periods, the survival of patients in this study during the late-period was high compared with that in previous reports, including reports from other countries (Supplemental Table 5) [4–9, 14–28]. Considering the outcome of patients who were treated with REBOA, their survival naturally varied by how REBOA was used and by the initiation timing of REBOA [16]. Therefore, after differentiating our analyzed patients from those who were dead on arrival, had unsurvivable injury or had underwent CPR (Supplemental Table 4), we could detect positive intervention effect of REBOA in study’s patients. This feature of our study could be one reason for the comparably high survival rate.

Interpretation and implications.

We could not conclude with certainty that REBOA was the only factor that contributed to improved survival because the survival of all trauma patients in Japan has increased during the time period of our study. To assess the association between REBOA and improved survival, we set the matched patients’ cohorts without REBOA, and we confirmed the matched patients’ outcome was not improved (Supplemental Table 2 and 6). While, other factors also contributed to improve the survival rate and we could control factors such as demographics and trauma severity to prevent indication bias, and we used mixed effects regression analysis to control biases that could result from differences in how REBOA was used in each hospital. Because the increased use of prehospital treatment was considered to be another confounder related to increased survival [29], we controlled it as a covariate. Early recognition of severely injured patients who need REBOA is important. After excluding the dead on arrival and CPR patients in this study, vital signs were more mild as they were expected and the patient could have underwent REBOA placement before becoming hemodynamically collapsed. The consideration to use REBOA as early as possible was an important indication bias in this study, therefore, we controlled for prehospital vital signs and therapeutic choices such as prehospital treatments and FAST. Early recognition and intervention for severely injured patients could contribute to increased survival of REBOA patients, however, we could not conclude from this study whether it was early recognition of severely injured patients or early intervention that improved survival. Matsumura, et al. reported from a Japanese REBOA registry that all prehospital REBOA patients survived [30]. We did not have information about prehospital REBOA use from the JTDB, but we believe that use of REBOA at the prehospital stage may be useful [14, 30]. On the other hands, earlier recognition and earlier placement of REBOA may imply that REBOA was placed in the patients who do not clinically need REBOA.

This study adds information about clinical treatment changes associated with the implementation of REBOA in Japan. REBOA was introduced in Japan in the early 2000s and its use has spread widely because the technique of endovascular therapy could match to emergency department physicians in Japan who had not enough experience of thoracotomy. As a result, clinicians selected trans-catheter arterial embolization (TAE) following REBOA as endovascular therapy. The selection of TAE was supported by dissemination of non-operative management, and the combination of TAE and REBOA could have contributed to improved survival [31, 32].

### Limitations

Our study has several limitations. First, because of the nature of the JTDB database, some clinically important information was not registered. For example, the JTDB had no information about plasma quantity. Use of the high ratio massive transfusion protocol is one area of progress in modern trauma care [33]. Besides, JTDB did not have information about tranexamic acid use. These confounders were thought to be also contributing the improved survival in this study. Second, some important variables had missingness to some degree, and missingness could affect the results of this study. Prehospital vital signs were thought to be related to the indication for REBOA, therefore, we used a multiple imputation method. Although our methodology in handling missing data was considered reasonable and statistically appropriate, the results should be interpreted with caution because of the missing data. Third, important limitation as to REBOA. JTDB had no information of indication of REBOA, REBOA balloon size, initiation of REBOA time, how to use of REBOA (partial or not), and or so. During the study periods, physicians skills of using REBOA and REBOA devices were also improved, these were important factors associated with improved survival.

## Conclusions

Survival of patients who were treated with REBOA during the late-period was improved compared with survival during the early-period, after adjustment for confounders. REBOA may be one of the important factors related to progression of modern trauma treatment.

## Supplementary information

**Additional file 1: Table S1.** Baseline characteristics and proportion of missing data in naïve dataset.

**Additional file 2: Table S2.** Subgroups’ and matched patients’ outcome according to year groups.

**Additional file 3: Table S3.** Survival of REBOA patients according to calendar year.

**Additional file 4: Table S4.** Survival of REBOA patients who had CPR according to calendar year.

**Additional file 5: Table S5**. Summary of previous studies about survival of REBOA patients.

**Additional file 6: Table S6.** Characteristics of matched patients with and without REBOA.

## Data Availability

The datasets generated during and/or analyzed during the current study are available from the corresponding author on reasonable request.

## Abbreviations

REBOA: Resuscitative endovascular balloon occlusion of the aorta
JTDB: Japan Trauma Data Bank
AIS: Abbreviated injury scale
ISS: Injury severity score
RTS: Revised trauma score
Ps: Probability of survival
CPR: Cardiopulmonary resuscitation
IQR: Interquartile range
VIF: Variance inflation factor
GCS: Glasgow coma scale
FAST: Focused assessment with sonography for trauma
CI: Confidence interval
OR: Odds ratio
