# Supplementary material for: Resuscitative endovascular balloon occlusion of the aorta may contribute to improved survival
Source: Scand J Trauma Resusc Emerg Med. 2020 Jun 30;28:62. doi: 10.1186/s13049-020-00757-2 (PMC7325257; doi:10.1186/s13049-020-00757-2)
Supplement: Supplementary file 1 — Additional file 1: Table S1. Baseline characteristics and proportion of missing data in naïve dataset. [file 13049_2020_757_MOESM1_ESM.docx]

Supplemental Table1. Baseline characteristics and proportion of missing data in naïve dataset

| Subgroups | Early-period (2004-2007)  n=91  Registered Missing, n (%) | | Mid-period (2008-2011)  n=276  Registered Missing, n (%) | | Late-period (2012-2015)  n=266  Registered Missing, n (%) | |
| --- | --- | --- | --- | --- | --- | --- |
| Age | 49 (30-68) | 0 (0) | 54 (32-7) | 0 (0) | 57 (36-72) | 0 (0) |
| Gender, Male (%) | 64 (70) | 0 (0) | 178 (65) | 0 (0) | 177 (67) | 0 (0) |
| Injury Type  TA  Fall  Other blunt  Penetrate | 55 (62)  23 (26)  5 (5.6)  6 (6.7) | 2 ((2.2) | 159 (59)  70 (26)  21 (7.8)  20 (7.4) | 6 (2.2) | 161 (61)  69 (26)  16 (6.0)  19 (7.2) | 1 (0.4) |
| Transport type  Ambulance  Dr-car  Helicopter  Other | 82 (91)  2 (2.2)  6 (6.7)  0 (0) | 1 (1.1) | 214 (81)  10 (3.8)  39 (15)  2 (0.8) | 11 (4.0) | 183 (70)  26 (9.9)  52 (20)  1 (0.4) | 4 (1.5) |
| Vital signs at prehospital  sBP  HR  RR | 98 (80-118)  94 (77-120)  24 (18-30) | 38 (42)  17 (19)  22 (24) | 94 (78-118)  96 (80-120)  24 (20-30) | 90 (33)  41 (15)  67 (24) | 107 (80-130)  100 (80-120)  24 (20-30) | 82 (31)  28 (11)  40 (15) |
| Vital signs at hospital arrival  sBP, mmHg  HR  RR  GCS | 80 (40-104)  108 (94-125)  24 (20-30)  9 (3-14) | 1 (1.1)  0 (0)  7 (7.7)  2 (2.2) | 80 (62-105)  105 (85-124)  27 (20-31)  11 (6-14) | 6 (2.2)  2 (0.7)  15 (5.4)  4 (1.4) | 80 (62-111)  109 (85-129)  25 (20-30)  12 (6-14) | 5 (1.9)  2 (0.8)  20 (7.5)  7 (2.6) |
| AIS, n, median (95%CI)  Head  Chest  Abdomen  Pelvis | 41, 3 (3-5)  52, 4 (3-4)  71, 4 (3-4)  51, 3 (2-5) | 0 (0) | 105, 4 (3-5)  162, 4 (3-4)  204, 3 (3-4)  171, 4 (3-5) | 0 (0) | 100, 4 (3-5)  166, 4 (3-4)  175, 4 (3-4)  175, 4 (3-5) | 0 (0) |
| ISS  RTS  Ps | 33 (20-45)  5.5 (3.8-6.9)  55 (18-91) | 2 (2.2)  7 (7.7)  10 (11) | 34 (20-45)  5.9 (4.4-7.1)  61 (23-89) | 1 (0.4)  22 (8.0)  26 (9.4) | 34 (25-47)  6.0 (4.5-7.1)  58 (23-88) | 7 (2.6)  28 (11)  36 (14) |
| FAST  Positive  Negative  Not conducted | 58 (65)  28 (32)  3 (3.3) | 2 (2.2) | 165 (62)  93 (35)  8 (3.0) | 10 (3.6) | 127 (50)  119 (47)  9 (3.5) | 11 (4.1) |
| Initial Treatment  Thoracotomy  Celiotomy  Damage control surgery  TAE | 3 (3.3)  52 (57)  16 (31)  25 (28) | 0 (0) | 16 (5.8)  143 (52)  63 (44)  69 (25) | 0 (0) | 13 (4.9)  114 (43)  36 (32)  89 (34) | 0 (0) |
| Blood Transfusion Quantity | 25 (16-40) | 11 (12) | 28 (16-46) | 78 (28) | 22 (13-38) | 72 (27) |

TA, traffic accident; sBP, systolic blood pressure; HR, heart rate; RR, respiratory rate; GCS, Glasgow Coma Scale; AIS, abbreviated injury scale; ISS, injury severity score; RTS, revised trauma score; Ps, provability of survival; FAST, focused assessment with sonography for trauma; TAE, transcatheter arterial embolization
