# Supplementary material for: Resuscitative endovascular balloon occlusion of the aorta may contribute to improved survival
Source: Scand J Trauma Resusc Emerg Med. 2020 Jun 30;28:62. doi: 10.1186/s13049-020-00757-2 (PMC7325257; doi:10.1186/s13049-020-00757-2)
Supplement: Supplementary file 2 — Additional file 2: Table S2. Subgroups’ and matched patients’ outcome according to year groups. [file 13049_2020_757_MOESM2_ESM.docx]

Supplemental Table 2. Subgroups’ and matched patients’ outcome according to year groups.

| Group | 2004-2007 | 2008-2011 | 2012-2015 | P-Value |
| --- | --- | --- | --- | --- |
| Severe head injury | 9 (29) | 20 (26) | 34 (42) | 0.112 |
| Severe abdominal injury | 18 (33) | 79 (56) | 78 (66) | <0.001 |
| Severe pelvis injury | 6 (20) | 46 (42) | 68 (54) | 0.002 |
| Matched patients with REBOA | 10 (27) | 55 (53) | 71 (62) | 0.001 |
| Matched patients without REBOA | 24 (63) | 61 (68) | 96 (76) | 0.197 |

REBOA, resuscitative endovascular balloon occlusion of the aorta
