# Supplementary material for: Resuscitative endovascular balloon occlusion of the aorta may contribute to improved survival
Source: Scand J Trauma Resusc Emerg Med. 2020 Jun 30;28:62. doi: 10.1186/s13049-020-00757-2 (PMC7325257; doi:10.1186/s13049-020-00757-2)
Supplement: Supplementary file 3 — Additional file 3: Table S3. Survival of REBOA patients according to calendar year. [file 13049_2020_757_MOESM3_ESM.docx]

Supplemental Table 3. Survival of REBOA patients according to calendar year

| Year | | | | | | | | | | | |
| --- | --- | --- | --- | --- | --- | --- | --- | --- | --- | --- | --- |
| 2004 | 2005 | 2006 | 2007 | 2008 | 2009 | 2010 | 2011 | 2012 | 2013 | 2014 | 2015 　 Annual P value  　　 Increase |
| In-hospital survival, n (%) | | | | | | | | | | | |
| 4 (27) | 6 (50) | 3 (20) | 22 (45) | 30 (47) | 29 (41) | 37 (54) | 38 (51) | 30 (49) | 46 (72) | 45 (63) | 39 (57) (3.0) 0.004 |

REBOA, resuscitative endovascular balloon occlusion of the aorta
