# Supplementary material for: Resuscitative endovascular balloon occlusion of the aorta may contribute to improved survival
Source: Scand J Trauma Resusc Emerg Med. 2020 Jun 30;28:62. doi: 10.1186/s13049-020-00757-2 (PMC7325257; doi:10.1186/s13049-020-00757-2)
Supplement: Supplementary file 4 — Additional file 4: Table S4. Survival of REBOA patients who had CPR according to calendar year. [file 13049_2020_757_MOESM4_ESM.docx]

Supplemental Table 4. Survival of REBOA patients who had CPR according to calendar year

| Subgroups | Early-period  (2004-2007)  n=38 | Mid-period  (2008-2011)  n=93 | Late-period  (2012-2015)  n=95 | P value |
| --- | --- | --- | --- | --- |
| Survival n (%) | 0 (0) | 1 (1.1) | 4 (4.2) | 0.205^*^ |

REBOA, resuscitative endovascular balloon occlusion of the aorta; CPR, cardiopulmonary resuscitation
