# Supplementary material for: Resuscitative endovascular balloon occlusion of the aorta may contribute to improved survival
Source: Scand J Trauma Resusc Emerg Med. 2020 Jun 30;28:62. doi: 10.1186/s13049-020-00757-2 (PMC7325257; doi:10.1186/s13049-020-00757-2)
Supplement: Supplementary file 5 — Additional file 5: Table S5. Summary of previous studies about survival of REBOA patients. [file 13049_2020_757_MOESM5_ESM.docx]

Supplemental Table 5

Summary of previous studies about survival of REBOA patients

| Study | Year | Design | Patient indication | Concept of study | Survival rate (%) |
| --- | --- | --- | --- | --- | --- |
| Gupta PK | 1989 | Single-center | REBOA patients with penetrating abdominal trauma | Descriptive | 7/21 (33) |
| Martinelli T | 1998-2007 | Single-center  retrospective | REBOA patients with pelvic fracture | Descriptive | 6/13 (46) |
| Brenner ML | 2012-2013 | Dual-center retrospective | REBOA patients | Descriptive | 4/6 (67) |
| Norii T | 2004-2011 | Multicenter  retrospective  (JTDB) | REBOA patients | Case control study  (propensity score matching) | 109/452 (24) |
| Saito N | 2007-2013 | Single-center  retrospective | REBOA patients with pelvic fracture or abdominal injury | Descriptive | 7/24 (29) |
| Moore LJ | 2012-2014 | Dual-center  retrospective | REBOA(+) vs RT with ACC | Descriptive? | 9/24 (37.5) |
| Inoue J | 2004-2014 | Multicenter  retrospective  (JTDB) | REBOA patients with surgery or TAE against torso trauma | Case control study  (propensity score matching) | 235/615 (38.2) |
| Tsurukiri J | 2010-2015 | Single-center  retrospective | REBOA patients | Descriptive | 6/16 (38) |
| Dubose JJ | 2013-2015 | Multicenter prospective  AORTA registry | REBOA(+) vs RT with ACC | Descriptive | 24/114 (21) |
| Teeter WA | 2014-2015 | Multicenter retrospective | REBOA patients | Descriptive | 14/33 (42) |
| Abe T | 2004-2013 | Multicenter  retrospective  (JTDB) | REBOA(+) vs RT with ACC | Case control study (propensity score matching) | 202/607 (33) |
| Matsumura Y | 2011-2015 | Multicenter retrospective | REBOA patients | Descriptive |  |
| Moore LJ | 2011-2015 | Multicenter retrospective | REBOA patients | Descriptive | 10/31 (32) |
| Aso S | 2010-2014 | Multicenter  retrospective  DPC database | REBOA(+) vs RT with ACC | Case control study (propensity score matching) | 101/191 (53) |
| Sadeghi M | 2011-2016 | Multicenter  retrospective | REBOA patients | Descriptive | 41/96 (43) |
| Brenner M | 2013-2017 | Single center  retrospective | REBOA patients | Descriptive | 23/79 (29) |
| Pieper A | 1996-2017 | Single center  retrospective | REBOA patients | Descriptive | 11/30 (37) |
| Brenner M | 2013-2017 | Multicenter  prospective | REBOA(+) vs RT with ACC | Case control study (univariate analysis) | 8/83 (9.6) |
| Sato R | 2013-2017 | Single center  retrospective | REBOA patients | Descriptive | 10/24 (41.7) |
| Ordonez CA | 2015-2016 | Single center  retrospective | REBOA patients with penetrating chest trauma | Descriptive | 6/7 (86) |
| Lendrum R | 2014-2018 | Single center  retrospective | Prehospital REBOA patient with pelvic fracture | Descriptive | 8/13 (62) |
| Joseph B | 2015-2016 | Multicenter  retrospective | REBOA patients | Case control study (propensity score matching | 90/140 (64) |

REBOA, resuscitative endovascular balloon occlusion of the aorta; JTDB, Japan Trauma Data Bank; RT, resuscitative thoracotomy; ACC, aortic cross-clamping; TAE, transcatheter arterial embolization; DPC, diagnosis procedure combination.
