# Supplementary material for: Resuscitative endovascular balloon occlusion of the aorta may contribute to improved survival
Source: Scand J Trauma Resusc Emerg Med. 2020 Jun 30;28:62. doi: 10.1186/s13049-020-00757-2 (PMC7325257; doi:10.1186/s13049-020-00757-2)
Supplement: Supplementary file 6 — Additional file 6: Table S6. Characteristics of matched patients with and without REBOA. [file 13049_2020_757_MOESM6_ESM.docx]

Supplemental Table 6. Characteristics of matched patients with and without REBOA.

| Variable | REBOA (+)  n=254 | REBOA (-)  n=254 | SD |
| --- | --- | --- | --- |
| Age | 56 (35-73) | 57 (36-72) | -0.8 |
| Sex, male (%) | 171 (67) | 167 (66) | 3.4 |
| Periods  Early  Mid  Late | 37 (15)  103 (41)  114 (45) | 38 (15)  90 (35)  126 (50) | -6.1 |
| Injury Type  TA  Fall  Other blunt  Penetrate | 158 (62)  57 (22)  20 (7.9)  19 (7.5) | 166 (65)  49 (19)  19 (7.5)  20 (7.9) | 3.0 |
| Transport Type  Ambulance  Dr-car  Helicopter | 205 (81)  19 (7.5)  30 (12) | 203 (80)  10 (3.9)  41 (16) | -7.6 |
| Prehospital vital signs  sBP  HR  RR | 100 (78-120)  98 (80-120)  24 (20-30) | 101 (84-123)  102 (83-120)  24 (21-30) | -8.9  -4.9  -13.4 |
| Vital signs at hospital arrival  sBP  HR  RR  GCS | 83 (67-110)  102 (83-120)  25 (20-30)  13 (6-14) | 86 (70-105)  104 (84-125)  25 (20-30)  13 (6-14) | 1.1  -1.9  -6.9  -7.3 |
| AIS, median  Head  Chest  Abdomen  Pelvis | 4 (3-5)  4 (3-4)  4 (3-4)  4 (3-5) | 4 (3-5)  4 (3-4)  4 (3-4)  3 (3-5) | 1.7  -10.1  -11.8  3.8 |
| Abdominal FAST  Positive  Negative  Not conducted | 144 (57)  103 (41)  7 (2.8) | 157 (62)  89 (35)  8 (3.1) | 8.6 |
| Initial treatment  Thoracotomy  Celiotomy  TAE | 8 (3.1)  125 (49)  82 (32) | 13 (5.1)  130 (51)  82 (32) | -11.2  -3.9  0 |
| Blood transfusion quantity | 24 (14-42) | 25 (10-42) | 3.9 |

REBOA, resuscitative endovascular balloon occlusion of the aorta; SD, standardized difference; TA, traffic accident; sBP, systolic blood pressure; HR, heart rate; RR, respiratory rate; GCS, Glasgow Coma Scale; AIS, abbreviated injury scale; FAST, focused assessment with sonography for trauma; TAE, transcatheter arterial embolization.
